# A Two-eRNA-Based Signature Can Impact the Immune Status and Predict the Prognosis and Drug Sensitivity of Lung Adenocarcinoma

**DOI:** 10.1155/2022/8069858

**Published:** 2022-05-10

**Authors:** Li Wang, Shao-quan Zhou, Yu Zhou, Jia-xi Lu

**Affiliations:** ^1^Department of Oncology, Chongqing General Hospital, Chongqing, China; ^2^Department of Radiology, Chongqing General Hospital, Chongqing, China; ^3^Department of Respiratory Critical Care Medicine, Chongqing Fuling People's Hospital, Chongqing, China

## Abstract

Enhancer RNAs (eRNAs) are intergenic long noncoding RNAs (lncRNAs) participating in the development of malignant cancers via targeting cancer-associated genes and immune checkpoints. Immune infiltration of the tumor microenvironment was positively associated with overall survival (OS) in lung adenocarcinoma (LUAD). In this study, we aimed to explore the clinical significance of PCBP1-AS1 in LUAD and developed a novel prognostic signature based on two eRNAs. Our team discovered that the expression of PCBP1-AS1 was distinctly downregulated in LUAD specimens compared with nontumor specimens. Lower PCBP1-AS1 expression was related to advanced clinical stages and poor prognosis. KEGG analysis unveiled that the coexpression genes of PCBP1-AS1 were involved in the regulation of several tumor-related pathways. In addition, remarkable associations were observed between the expression of PCBP1-AS1 and the levels of several immune cells. Then, we used PCBP1-AS1 and TBX5-AS1 to develop a prognostic model. Survival assays unveiled that patients with higher risk scores exhibited a shorter OS in contrast to patients with lower risk scores. In addition, multivariable Cox regressive analysis indicated that the risk score was an independent prediction factor in LUAD sufferers. The anticancer drug sensitivity analysis indicated that risk score had a positive relationship with several anticancer drugs. Taken together, our findings indicated PCBP1-AS1 as a function modulator in LUAD development. In addition, we constructed a robust immune-related eRNA signature which might be a clinical prognosis factor for LUAD patients.

## 1. Introduction

Lung cancer, as the leading cause of tumor-related mortality, is still a severe public healthcare challenge across the world [1]. Tumor epidemiologic data revealed that, in 2012, there were approximately 1.8 million novel pulmonary carcinoma patients and 1.6 million mortalities, separately, representing approximately 13% of the sum of tumor cases and 20% of the sum of tumor mortalities, respectively [2, 3]. Lung adenocarcinoma (LUAD) is the predominant subtype of pulmonary carcinoma [4]. The 5-year OS of pulmonary carcinoma sufferers can attain 55.2%. Nevertheless, over half of pulmonary carcinoma sufferers are diagnosed in late periods with a 5 − year OS < 20%, even with the fast advancement of diagnoses and therapies [5, 6]. As LUAD sufferers represent the majority of pulmonary carcinoma sufferers, it is imperative to identify LUAD biomarkers with higher sensitiveness and specificness.

With the progress of the second- and third-generation sequence identification techniques, ncRNAs have aroused remarkable interest because of the capability of regulating genetic expression [7]. eRNA is a kind of ncRNA transcribed from the enhancer [8]. Substantial eRNAs have been identified to be vital for the transcriptional process of mankind cells [9]. Mounting data have revealed that lncRNAs participate in almost all biology processes. Increasing evidence points to functional roles of at least a subset of eRNAs in gene regulation in both normal and cancer cells, adding new insights into the action mechanisms of enhancers [10, 11]. In the progression of malignancies, eRNAs can be involved in the expressions of tumor genes and the stimulation of oncogenesis pathways [12, 13]. For instance, the expression of eMARC1 was reported to be high in bladder cancer samples and lineage cells, and eMARC1 overexpression facilitated the development of bladder cancer cells, whereas the knockout of eMARC1 repressed tumor genesis [14]. Shang et al. reported that SLC2A1-AS1 was often regulated downward in hepatocellular carcinoma specimens. Deleting SLC2A1-AS1 was remarkably related to relapse-free survival in hepatocellular carcinoma. SLC2A1-AS1 overexpression remarkably repressed proliferative and metastatic activities in hepatocellular carcinoma via the transcription suppression of GLUT1 [15]. Moreover, several eRNAs have been reported to be related to the prognoses of several tumors, including LUAD patients [16, 17]. Intriguingly, mounting proofs have revealed the direct or nondirect interplay between eRNAs and immunity status in the LUAD microenvironment, despite the fact that the potential causal links are still elusive [18, 19].

The abnormal expressions of eRNAs and aberrant variations are commonly seen in oncocytes and are related to cancer development [20, 21]. Nevertheless, the direct interplay and effects on LUAD are elusive and warrant more explorations. Our team aimed to first investigate the expression and clinical significance of eRNAs in LUAD by biological information approaches and find biomarkers with potential utilization value so as to improve the clinical efficacy and improve the prognosis of LUAD patients.

## 2. Materials and Methods

### 2.1. Data Acquiring and Cleaning

LUAD sufferers' transcriptomic sequence data and clinic data were acquired from the UCSC Xena TCGA LUAD cohort. After deleting LUAD samples with low-quality data, 513 LUAD patients were used for survival assays. The variant profiles were obtained from the TCGA database via R program 4.0.0 with the package TCGAbiolinks. Data cleaning was completed via the R program.

### 2.2. Identifying Candidate eRNAs in LUAD

lncRNAs transcripted from active tissue-specific enhancers and the potential targeted genes were explored via Predicting Specific Tissue Interactions of Genes and Enhancers (PreSTIGE, https://galaxyproject.org/use/prestige/). Then, BioMart (https://http://www.ensembl.org/) was used to realize the conversion of the Ensembl transcript ID into a genetic symbol for further analyses. The prognosis-related eRNAs were screened by Kaplan-Meier (K-M) analyses. OS (*p* < 0.05) were considered candidate eRNAs in LUAD.

### 2.3. Gene Set Enrichment Analysis (GSEA)

GSEA was completed on the normalised RNA sequencing data, which was acquired from the TCGA database. The GO terms and KEGG pathways were utilized to explore the potential biofunctions of PCBP1-AS1. A false discovery rate (FDR) < 0.050 and a nominal *p* < 0.050 had significance on statistics.

### 2.4. Difference Analysis of Scores with Clinical Stages

The clinicopathologic feature data in correspondence to LUAD specimens were acquired from TCGA. The analyses were completed by R language and Wilcoxon rank sum or Kruskal-Wallis rank sum test as the significance test relying on the quantity of clinical phases for contrast.

### 2.5. Tumor Immune Microenvironment Analyses

CIBERSORT was utilized to speculate the relative fractions of 22 infiltration immunocyte types in every cancer specimen via R package [22]. The ESTIMATE arithmetic was leveraged to obtain the immunity scoring for every specimen [23].

### 2.6. Construction of Prognostic Immune Gene Signature

Our team tried to design a prognosis-related multiple immunogene hallmark based on PCBP1-AS1 and TBX5-AS1. The stepwise variable selection was completed via the Akaike Information Criterion in Cox models. Posterior to the selection of immunogenes, the prognosis indicator, namely, risk scoring, was produced: risk scoring = A1∗B1 + A2∗B2 + ⋯+AiBi, where A1 was the expressing level of every gene and B1 was the risk coefficient of every gene originated from the Cox model. K-M curves, log-rank tests, and univariable Cox analysis were used to evaluate the relationship of the immunity-associated genetic hallmark and clinic features with OS. Multivariable analyses were completed for the risk scores with modification for age, sex, and clinical phase. The time-reliant receiver operating characteristic (ROC) curves were utilized to identify the prognosis accurateness of the risk scores via the survival ROC package.

### 2.7. Statistical Analysis

The entire analysis was completed via R 3.4.3 (R Core Team, Massachusetts, USA). Continuous variates were studied via the *t*-test or the Wilcoxon rank sum test, and categorical variates were contrasted with the Pearson chi-square test. When the midvalue of risk scores in every dataset was utilized as a cut-off to contrast survival risks between the risk_high_ group and the risk_low_ group, a K-M curve was plotted. To determine independent risk factors for OS, multivariable Cox regressive analyses were completed to modify covariables. The immunity scores were computed via the ESTIMATE package. Time-dependent ROC curves were done with timeROC package. Differences were considered statistically significant when *p* was less than 0.05.

## 3. Results

### 3.1. The Distinct Upregulation of PCBP1-AS1 in LUAD

**Figure 1:**
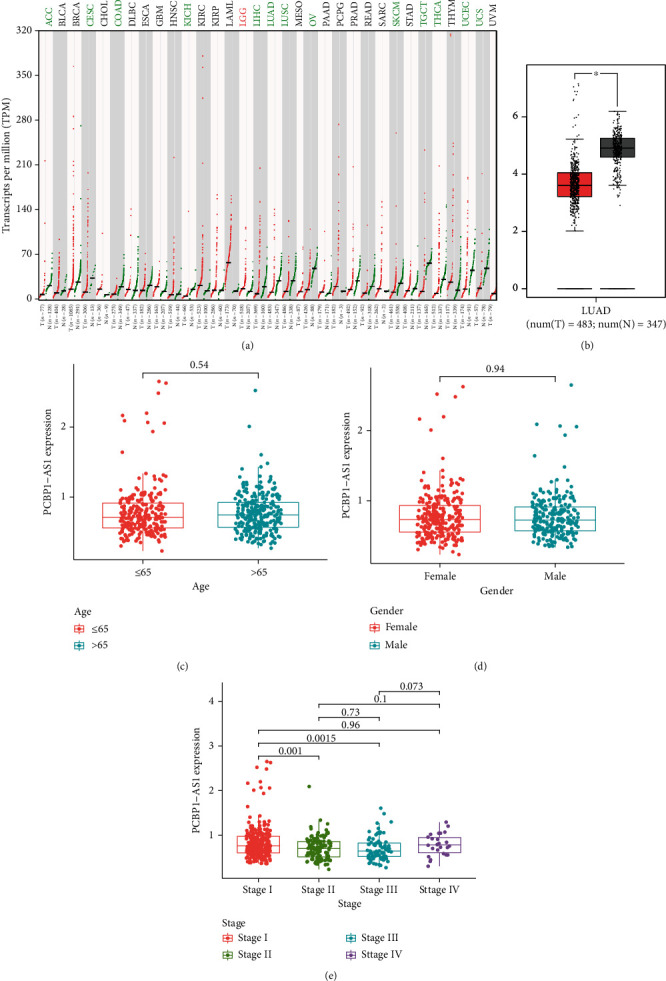
PCBP1-AS1 expression was increased in LUAD patients. (a) PCBP1-AS1 expression in 33 types of cancer on the foundation of TCGA datasets. (b) The distinct upregulation of the expression of PCBP1-AS1 in LUAD samples and healthy pulmonary samples. (c, d) There were no distinct associations between PCBP1-AS1 expression and age and gender. (e) The different levels of PCBP1-AS1 in LUAD specimens with different clinical stages. ^∗^*p* < 0.05.

For the sake of investigating the potential roles of PCBP1-AS1 in tumor progression, our team retrieved GEPIA [24], and the pancancer expressions of PCBP1-AS1 are shown in Figure 1(a) which revealed that the dysregulation of PCBP1-AS1 levels in tumors may be a frequent event. Importantly, our team discovered that the expressions of PCBP1-AS1 were remarkably reduced in LUAD samples in contrast to nontumor lung samples (Figure 1(b)). Then, we analyzed the expressing pattern of PCBP1-AS1 in LUAD specimens with different clinical features. No distinct relationship existed between the PCBP1-AS1 expression and age and sex (Figures 1(c) and 1(d)). However, we found that LUAD specimens with late period phases displayed a decreased expression of PCBP1-AS1 compared with samples with early phases (Figure 1(e)).

### 3.2. The Prognostic Value of PCBP1-AS1 in LUAD Patients

**Figure 2:**
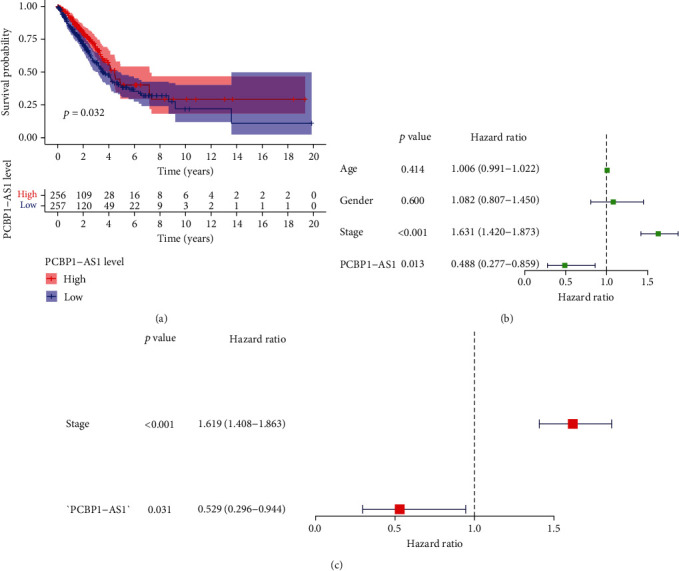
The prognosis significance of the expressions of PCBP1-AS1 in LUAD sufferers. (a) Survival probability of 512 LUAD patients separated into 2 groups on the foundation of the mean expression of PCBP1-AS1. (b, c) Univariable and multivariable analyses of OS in LSCC sufferers.

To identify the correlation of PCBP1-AS1 levels with the prognoses of LUAD, we divided sufferers into 2 groups as per the expressing levels of PCBP1-AS1. As expected, K-M analyses revealed that high expressions of PCBP1-AS1 predicted remarkably better OS (*p* = 0.032, Figure 2(a)). Moreover, we further performed COX analyses for further identification of the effects of PCBP1-AS1 used as a potential biomarker for LUAD patients. Univariate analysis showed that clinical stage and PCBP1-AS1 expression levels were significantly related to overall survival (Figure 2(b)). Multivariate analysis showed that clinical stage (HR = 1.408, *p* < 0.001) and PCBP1-AS1 expression levels (HR = 0.529, *p* = 0.031) were independent prognostic factors (Figure 2(c)).

### 3.3. GO Annotation and KEGG Pathway Enrichment Analysis

**Figure 3:**
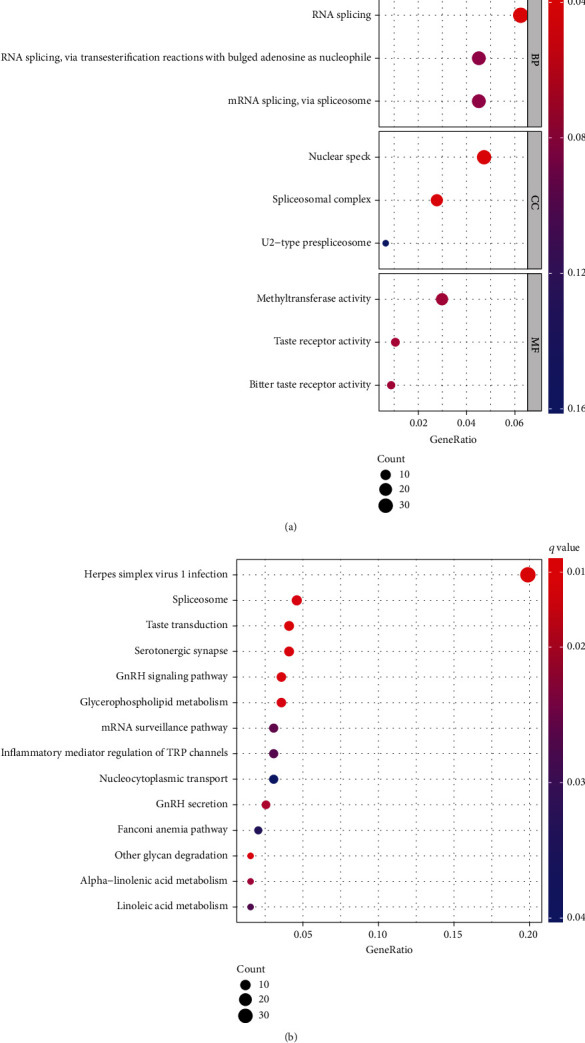
Function enrichment analyses of 2183 genes involved in the levels of PCBP1-AS1: (a) GO enrichment analysis; (b) KEGG pathway enrichment analysis.

For the purpose of further revealing the biofunctions of PCBP1-AS1 in LUAD, we screened the related genes of PCBP1-AS1. As shown in Table S1, an overall 2183 transcripts displayed a remarkable association with PCBP1-AS1 (*p* < 0.05). GO enrichment analyses and KEGG pathway analyses of the 2183 targeted genes offered the foundation to biologically research those genes. GO BP analysis revealed that 2183 genes were markedly enriched in the RNA splicing through transesterification reaction with bulged adenosine as the nucleophile and mRNA splicing via spliceosomes. For GO CC analysis, the significantly enriched terms were nuclear speck, spliceosome complexes, and U2-type prespliceosome. The significantly enriched MF terms included methyltransferase, taste acceptor, and bitter taste acceptor activities (Figure 3(a)). In addition, the markedly enriched pathways for 2183 genes were Herpes simplex virus 1 infection, spliceosome, GnRH signal path, and inflammation mediator modulation of TRP channels (Figure 3(b)).

### 3.4. Correlation of PCBP1-AS1 with the Level of Tumor-Infiltrating Immune Cells (TICs)

**Figure 4:**
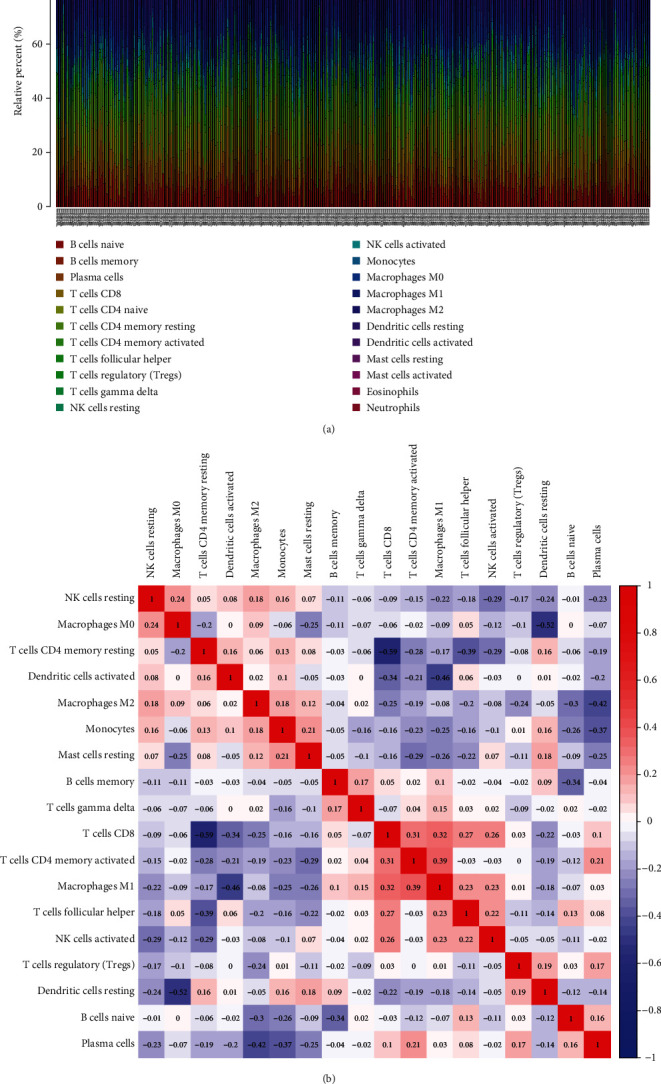
TIC profiles in LUAD samples and association assay. (a) Visualisation of the infiltration levels of several immunocytes in the LUAD specimens and nontumor samples. (b) The association among all immune cells.

**Figure 5:**
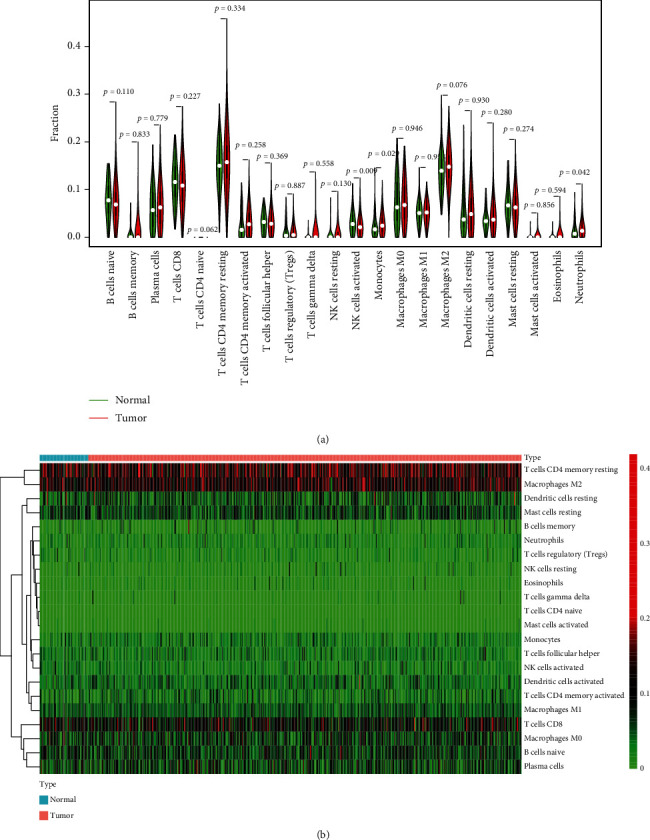
(a, b) Difference in the proportion of 22 TIICs in LUAD samples and nontumor samples.

**Figure 6:**
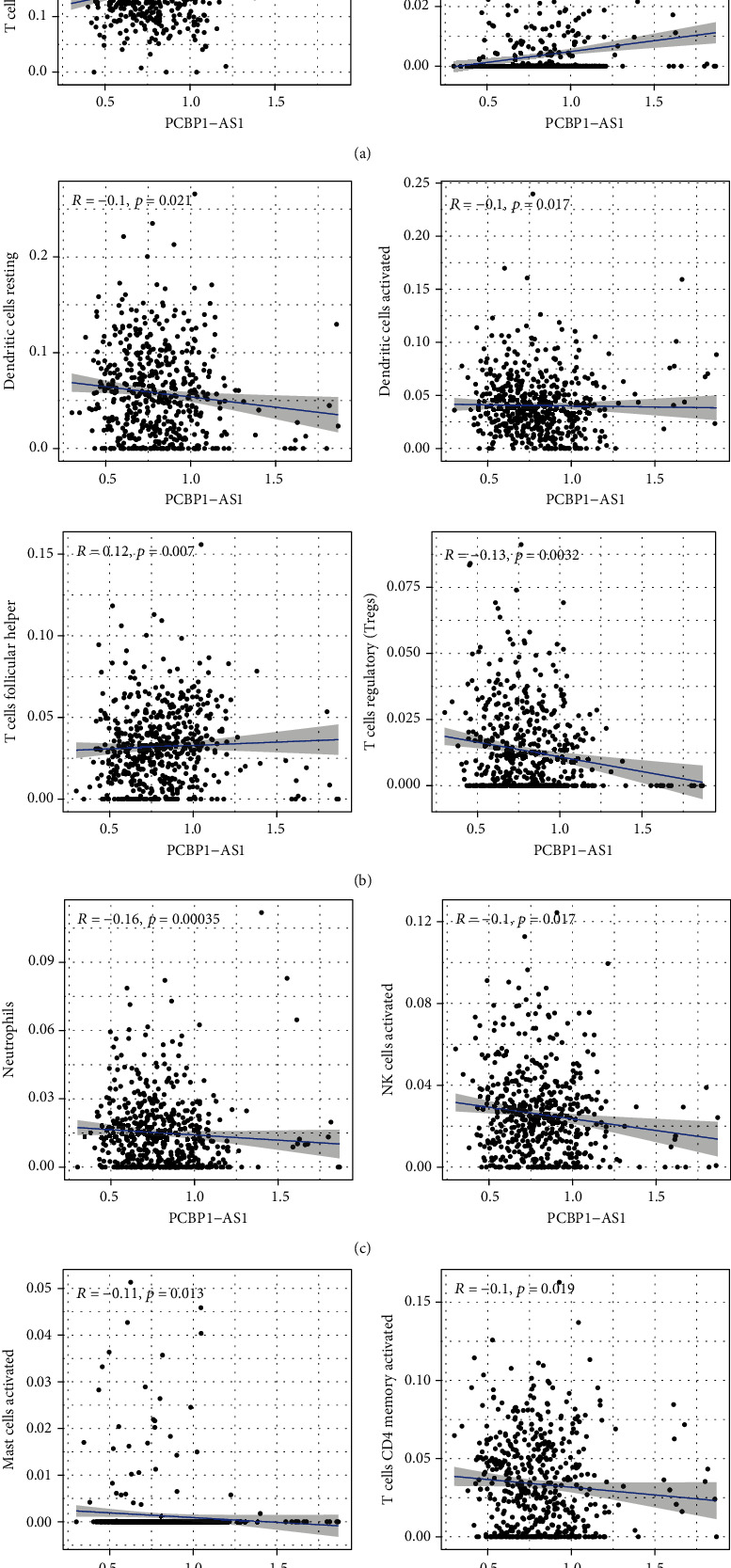
Relationship of TIC level with the expression of PCBP1-AS1. Dispersed point chart presented the relationship of 10 types of TIC level with the expression of PCBP1-AS1 (*p* < 0.05), like (a) CD4 memory stimulated T cells and resting NK cells; (b) resting DCs and stimulated DCs; (c) neutrophilic cells, stimulated NK cells, Tfh, and Tregs; (d) stimulated mastocytes and CD4 memory resting T cells. The association assay was completed via Pearson coefficient.

To substantiate the relationship between the expressions of PCBP1-AS1 and immunity microenvironment, the level of TIC subsets was studied via CIBERSORT arithmetic, and 21 types of immunocyte profiles in LUAD sufferers were obtained (Figures 4(a) and 4(b)). We observed that the levels of monocytes, neutrophils, activated NK cells were abnormal in LAUD specimens (Figures 5(a) and 5(b)). In addition, remarkable associations were observed between the expressions of PCBP1-AS1 and the levels of stimulated DCs, resting DCs, stimulated mastocytes, neutrophilic cells, stimulated NK cells, resting NK cells, CD4 memory stimulated T cells, CD4 memory resting T cells, follicular helper T cells (Tfh), and regulatory T cells (Tregs) (Figures 6(a)–6(d)). Those outcomes substantiate the roles of the expressions of PCBP1-AS1 in the immunoactivity of TME.

### 3.5. TBX5-AS1 Expression Was Decreased in LUAD

**Figure 7:**
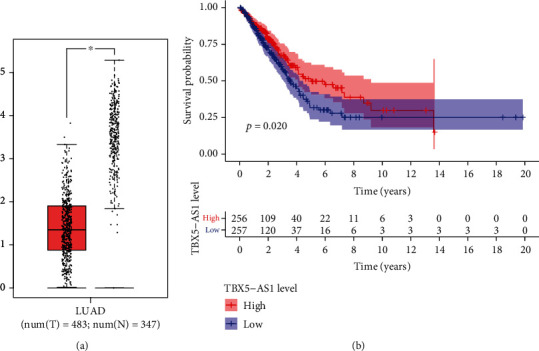
The expressions and clinical value of TBX5-AS1 in LUAD patients. (a) The expression of TBX5-AS1 was decreased in LUAD samples in contrast to nontumor samples. (b) High expression of TBX5-AS1 correlated with poor OS based on the TCGA.

**Figure 8:**
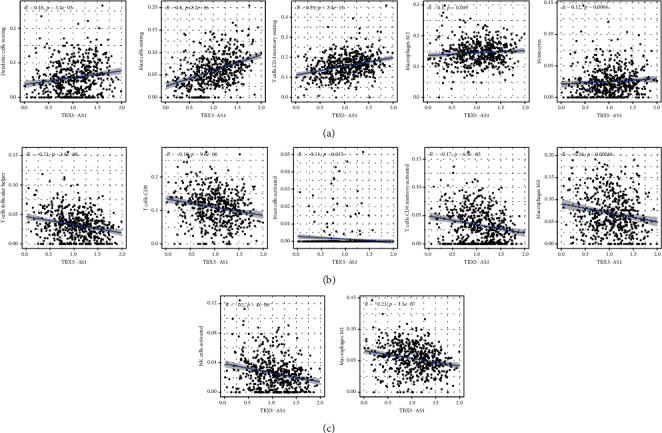
Association of TIC level with the expression of PCBP1-AS1. (a–c) Scatter plot presented the association of 12 types of TIC proportion with the PCBP1-AS1 expressions.

To further develop a novel prognostic model based on eRNAs, we screened an eRNA and focused on TBX5-AS1. Previously, TBX5-AS1 has been discovered to participate in the tumorous development of several cancers, including LUAD [16, 25, 26]. In addition, its dysregulation was also reported. Herein, our team studied TCGA datasets and discovered that the expression of TBX5-AS1 was distinctly decreased in LUAD specimens (Figure 7(a)). Moreover, sufferers with higher expressions of TBX5-AS1 exhibited a better OS in contrast to sufferers with lower expressions of TBX5-AS1 (*p* = 0.020, Figure 7(b)). Moreover, we observed that remarkable associations existed between the expression of TBX5-AS1 and the levels of resting DCs, Macrophagus M0, Macrophagus M1, Macrophagus M2, stimulated mastocytes, resting mastocytes, mononuclear cells, stimulated NK cells, CD4 memory stimulated T cells, CD4 memory resting T cells, CD8 T cells, and Tfh, highlighting its involvement in immune function (Figures 8(a)–8(c)).

### 3.6. Construction and Verification of Immune-Associated eRNA Signature

**Figure 9:**
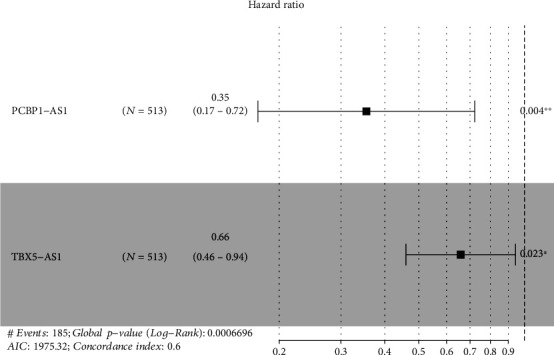
Multivariate analysis of OS in LUAD sufferers.

**Figure 10:**
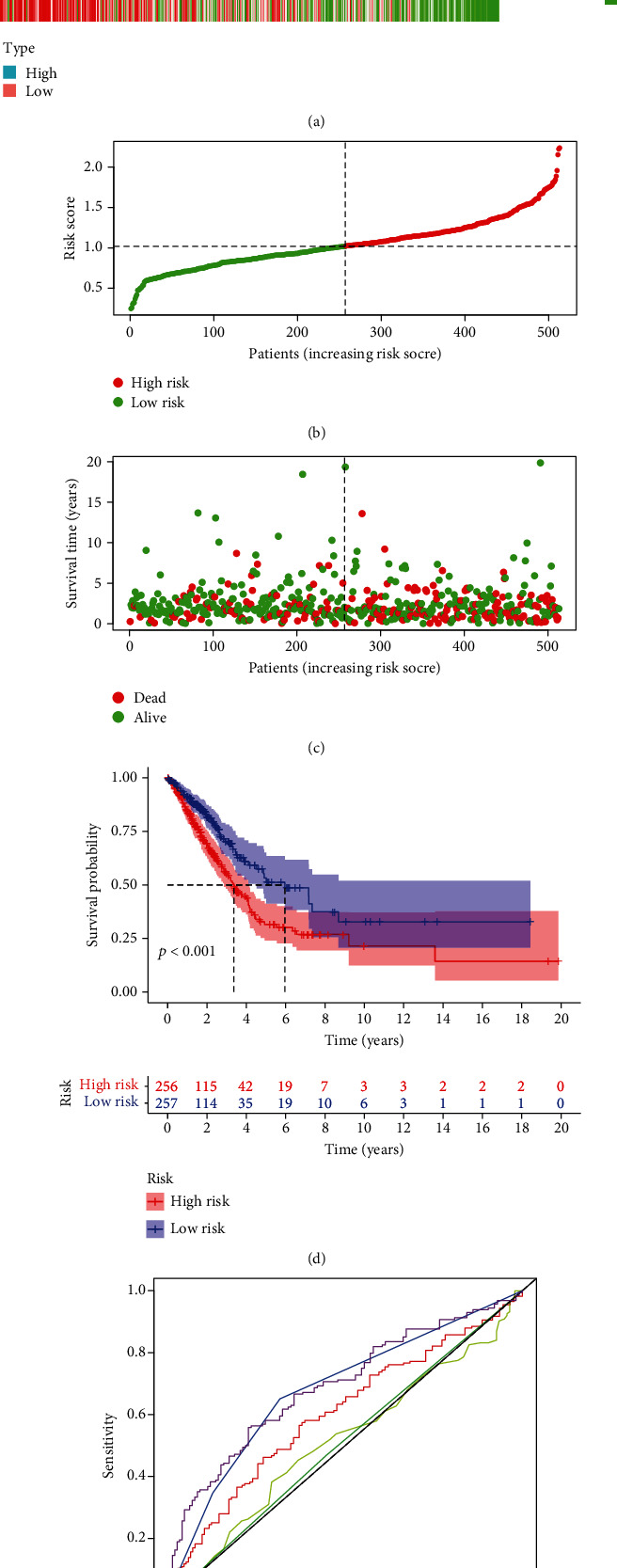
A novel prognostic model of LUAD sufferers. (a) The expression pattern of PCBP1-AS1 and TBX5-AS1 in all LUAD specimens. (b) The prognostic model distribution of LUAD patients. (c) The OS of sufferers in the TCGA dataset. (d) The K-M curve represented that risk_high_ sufferers displayed remarkably poorer OS in contrast to risk_low_ sufferers. (e) ROC curve of risk scores and the rest of clinical factors.

**Figure 11:**
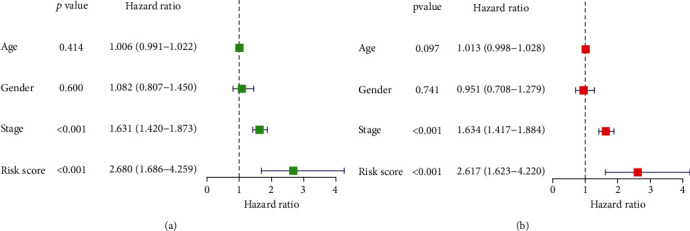
Independent prognosis analyses of risk scoring and clinic parameters: (a) univariable Cox regressive analyses; (b) the multivariable Cox regressive analyses.

We used TBX5-AS1 and PCBP1-AS1 to develop establishment of a prognosis model via the risk scoring = (−1.0372∗expressing level of PCBP1 − AS1) + (−0.4178∗expressing level of TBX5 − AS1) (Figure 9). The mean risk scores were utilized to separate all LUAD sufferers into the risk_high_ and risk_low_ groups. The expression details of TBX5-AS1 and PCBP1-AS1 are shown in Figure 10(a). The distributional status of risk scores and survival status are presented by Figures 10(b) and 10(c). The K-M analysis revealed that the OS of the risk_high_ patients was remarkably worse in contrast to that of the risk_low_ patients (Figure 10(d)). The area under the time-reliant ROC curves (AUCs) for 5-year OS was 0.603, revealing a satisfactory prediction power of such prognosis model (Figure 10(e)). Moreover, univariate analyses revealed that phase and risk scores were distinctly associated with overall survival (Figure 11(a)). More importantly, multivariate analysis demonstrated that phase and risk scores were independent prognosis factors (Figure 11(b)).

### 3.7. Correlation Analysis between Risk Score and Drug Sensitivity

**Figure 12:**
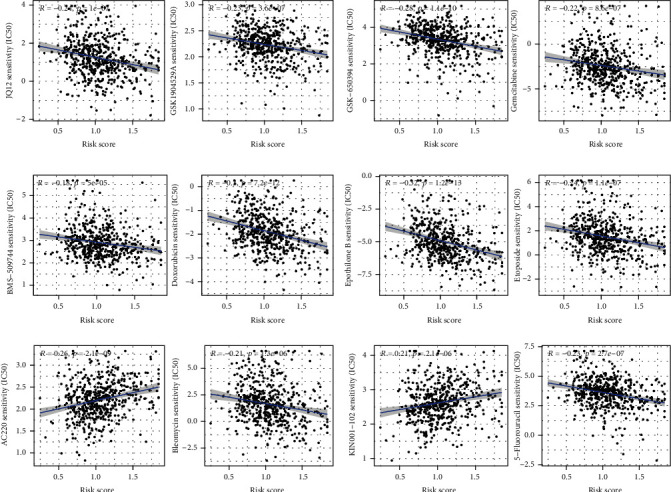
Correlation analysis between risk score and drug sensitivity of anticancer drugs.

Finally, we analyzed the possible association between risk score and drug sensitivity. As shown in Figure 12 and Figure S1, we observed that the risk score was associated with the sensitivity of many anticancer drugs, such as 5-fluorouracil, AC220, bleomycin, BMS−509744, doxorubicin, epothilone B, etoposide, gemcitabine, GSK-650394, GSK1904529A, JQ12, KIN001-102, KIN001-135, midostaurin, mitomycin C, NG-25, obatoclax mesylate, OSU-03012, PF-562271, phenformin, pyrimethamine, rapamycin, thapsigargin, tipifarnib, and TL-2-105. Our findings firstly analyzed the relationships between risk scores with the drug sensitivity of anticancer drugs.

## 4. Discussion

As per the immune editing theory, the progression of immunoescape causal links in neoplasm damage enables oncocytes to survive the original stage of cancer eradication reliant on the stimulation of anticancer immune activity [27, 28]. The change in the immunoresponse from anticancer status to cancer-tolerant status facilitates the LUAD development [29]. Immunocytes and immunogenes have been discovered as new prognostic markers and treatment targets for LUAD [30, 31]. Herein, our team strived to establish a new immunity-associated eRNA prognosis hallmark of LUAD, to characterise the eRNA hallmark as a promising prognosis tool, and to determine treatment targets for LUAD.

Many studies have reported the vital effects of eRNAs on the development of various cancers [26, 32]. Herein, our team studied TCGA datasets and identified many survival-associated eRNAs. Then, we focused on PCBP1-AS1 whose expression was low in LUAD samples. Importantly, the low expressions of PCBP1-AS1 were related to advanced stages and poor prognosis. Recently, the effects of PCBP1-AS1 have been studied in multiple tumors. Zhang et al. found that higher expressions of PCBP1-AS1 might reveal poorer prognoses for sufferers and might reinforce the deubiquitination of AR/AR-V7 via stabilising the USP22-AR/AR-V7 complexes, hence avoiding AR/AR-V7 degradation via the ubiquitin-proteasome channel [33]. Luo and his group revealed that PCBP1-AS1 was highly expressed in human hepatocellular carcinoma and markedly related to unsatisfactory prognoses in sufferers with hepatocellular carcinoma. The knockout of PCBP1-AS1 suppressed the proliferation, migration, and invasion of HCC cells through modulating PCBP1/PRL-3/AKT signal path [34]. Previously, the expression of PCBP1-AS1 was also discovered to be low in LUAD, which was consistent with our findings [35]. More importantly, we firstly provided evidence that PCBP1-AS1 was an independent prognosis factor for 5-year OS in LUAD patients, suggesting it as a novel biomarker for LUAD patients.

Herein, GO and KEGG outcomes reveal that PCBP1-AS1 was related to several signal paths, like the GnRH signaling pathway, glycerophospholipid metabolism, Herpes simplex virus 1 infection, and serotonergic synapse. These findings suggested that PCBP1-AS1 may play an important role via regulating the above tumor-related pathways. In recent years, many kinds of LUAD have been identified to be immunogenic and sponged in cancer-infiltrating lymph cells [36]. Several discoveries indicated that DRP1 exerted an effect on cancer-immunity interplay and that the expressions of PCBP1-AS1 were related to the levels of immunity infiltration in LUAD. Our team explored the association between the expressions of PCBP1-AS1 and immunity infiltration in LUAD via CIBERSORT. Intriguingly, our team discovered that the expressions of PCBP1-AS1 were related to the infiltrative activities of diverse immunocytes, like activated DCs, resting DCs, activated mast cells, neutrophils, activated NK cells, CD4 memory resting T cells, Tfh, Tregs, resting NK cells, and CD4 memory activated T cells. Tumor-infiltrating immune cells have been confirmed to be related to the clinical outcome of LUAD sufferers [37, 38]. Our findings suggested that PCBP1-AS1 might affect the prognoses of LUAD sufferers via modulating TICs.

The available largescale genetic expression profiles enable us to determine more dependable prognosis hallmarks in a variety of tumors [39, 40]. Some researches have put forward genetic expression prognosis hallmarks in LUAD [41, 42]. To further develop a novel prognostic model based on eRNAs, we further focused on TBX5-AS1 which also exhibited a distinct upregulation in LUAD and predicted a good prognosis [16]. Interesting, it is also an immune-related eRNA and was related to the infiltrative activities of diverse immunocytes. Then, we used the Cox model to examine risk scoring which was utilized to separate the entire sufferers into two groups. The elevating percentage of mortality usually occurs in risk_high_ sufferers. In Kaplan-Meier analysis, risk_high_ sufferers presented poorer OS, in contrast to risk_low_ sufferers. The results of multivariable Cox regressive analysis verified that risk scoring was an independent prognosis factor for the 5-year OS of LUAD patients. Our findings suggested that such a hallmark could be a selection tool for risk_high_ sufferers in following molecule biology researches and be a valid tool for doctors to forecast prognoses.

There was a strong correlation between risk scores and anticancer medication sensitivity in our study; we first established this correlation. We observed that the risk score was associated with the sensitivity of many anticancer drugs, such as 5-fluorouracil, AC220, bleomycin, BMS-509744, doxorubicin, epothilone B, etoposide, gemcitabine, GSK-650394, GSK1904529A, JQ12, KIN001-102, KIN001-135, midostaurin, mitomycin C, NG-25, obatoclax mesylate, OSU-03012, PF-562271, phenformin, pyrimethamine, rapamycin, thapsigargin, tipifarnib, and TL-2-105. Risk scores and the sensitivity of anticancer medications were first examined in our study, which provided a fresh insight into the treatment of tumors and the prevention of tumor resistance [43, 44].

There are certain flaws in the present work that should be noted. First, although the dysregulated eRNAs were associated with LUAD, no additional experiments were conducted to validate these findings. Second, this paper was finished retrospectively, and more prospective clinic datasets are required to substantiate the outcome herein. Third, the hallmark in this work was established by 2 genes; more biofunctions need to be further investigated in LUAD.

## 5. Conclusions

Holistically, we were the first team to explore the prognostic significance of eRNAs in LUAD. An eRNA signature was established to forecast prognoses for LUAD sufferers. Nevertheless, the potency of the risk scoring signatures requires further tests in larger cohorts of LUAD sufferers, and exploration of the molecule-level causal links of genes in the signature is warranted in future studies.

## Data Availability

The data used to support the findings of this study are available from the corresponding author upon request.

## Supplementary Materials

Supplementary 1 Figure S1: correlation analysis between risk score and drug sensitivity of anticancer drugs.

Supplementary 2 Table S1: identification of survival-related eRNAs in LUAD based on TCGA datasets.
