# Supplementary figures and images for: A Two-eRNA-Based Signature Can Impact the Immune Status and Predict the Prognosis and Drug Sensitivity of Lung Adenocarcinoma
Source: J Immunol Res. 2022 May 10;2022:8069858. doi: 10.1155/2022/8069858 (PMC9115606; doi:10.1155/2022/8069858)

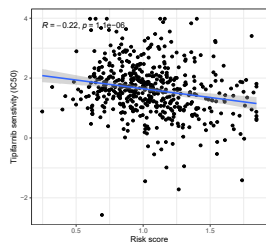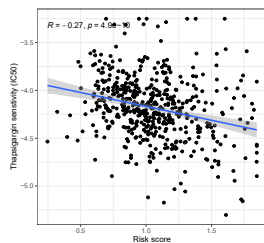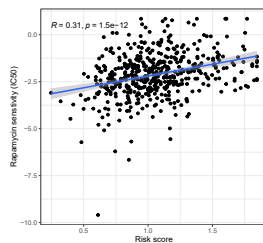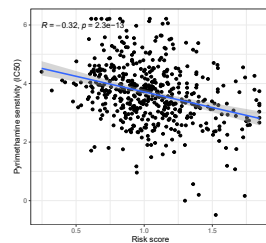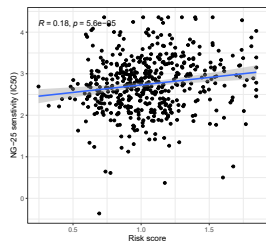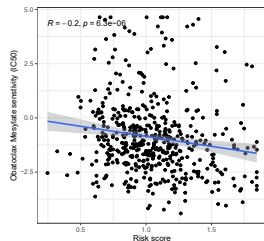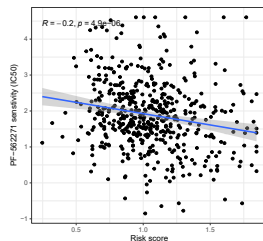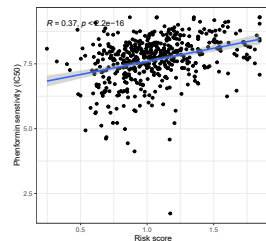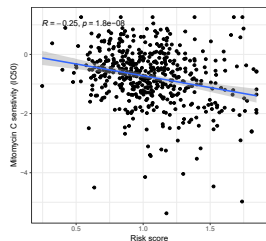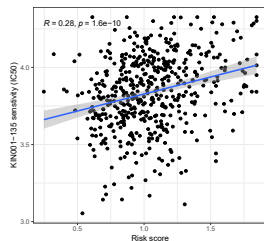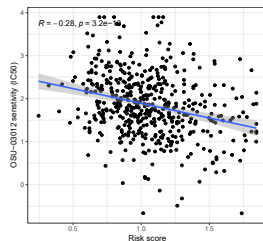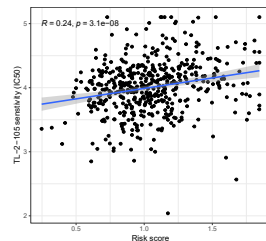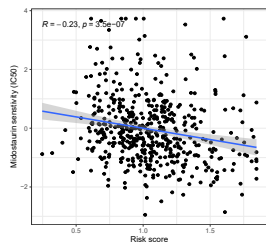

Supplement: Supplementary 1 — Figure S1: correlation analysis between risk score and drug sensitivity of anticancer drugs. [file 8069858.f1.pdf]
