# Supplementary material for: A Two-eRNA-Based Signature Can Impact the Immune Status and Predict the Prognosis and Drug Sensitivity of Lung Adenocarcinoma
Source: J Immunol Res. 2022 May 10;2022:8069858. doi: 10.1155/2022/8069858 (PMC9115606; doi:10.1155/2022/8069858)
Supplement: Supplementary 2 — Table S1: identification of survival-related eRNAs in LUAD based on TCGA datasets. [file 8069858.f2.docx]

Table S1. Identification of survival-related eRNAs in LUAD based on TCGA datasets.

| gene | KM |
| --- | --- |
| ITFG2-AS1 | 0.029931 |
| AL136369.2 | 0.034828 |
| AP002992.1 | 0.036257 |
| AC010343.3 | 0.003111 |
| LINC01615 | 0.039712 |
| LINC02812 | 0.002828 |
| DRAIC | 0.002097 |
| PCBP1-AS1 | 0.032138 |
| IFNG-AS1 | 0.036002 |
| GAS1RR | 0.00058 |
| LINC02613 | 0.000714 |
| NBDY | 0.003373 |
| LINC02611 | 0.004212 |
| LINC02044 | 0.020954 |
| LINC01322 | 0.015486 |
| AL359710.1 | 0.019461 |
| AP5B1 | 0.034221 |
| MIR583HG | 0.030284 |
| AL031846.1 | 0.034321 |
| AC084375.1 | 0.014973 |
| LINC02754 | 0.014297 |
| LINC00518 | 3.28E-05 |
| AL606491.1 | 0.004539 |
| LINC02757 | 0.015363 |
| LINC02036 | 0.008884 |
| AC090023.2 | 0.014145 |
| LINC01150 | 0.008059 |
| AL158835.1 | 0.000618 |
| MIR4435-2HG | 0.009491 |
| AC005013.1 | 0.030442 |
| AP003721.2 | 0.040072 |
| LRRC37A11P | 0.005117 |
| AC023796.1 | 0.003747 |
| AL031289.1 | 0.048608 |
| GCC2-AS1 | 0.004488 |
| LINC00926 | 0.043636 |
| PINLYP | 0.011707 |
| AL138767.3 | 0.041641 |
| LHFPL3-AS2 | 0.002031 |
| AL390778.2 | 0.04255 |
| AL445430.2 | 0.000505 |
| CRNDE | 0.000844 |
| LINC01238 | 0.034148 |
| PRDM16-DT | 0.000299 |
| AL451069.1 | 0.001799 |
| TBX5-AS1 | 0.019877 |
| AC106795.1 | 0.011967 |
| FAM21EP | 0.002124 |
| AC006357.1 | 0.006043 |
| AC093911.1 | 0.026562 |
| OSMR-AS1 | 0.043023 |
| AL139383.1 | 0.01398 |
| AC128709.2 | 0.006204 |
| AC009226.1 | 0.019659 |
| LNCAROD | 0.031311 |
| BAALC-AS1 | 0.044631 |
| AL772337.2 | 0.013262 |
| AL691447.2 | 0.010661 |
| LINC02422 | 0.008198 |
| RAB30-DT | 0.029679 |
| UCA1 | 0.01377 |
| AL445430.1 | 0.015814 |
| AL137025.1 | 0.023365 |
| AC004923.1 | 0.008951 |
| LINC02577 | 0.015963 |
| TMEM210 | 0.003174 |
| AC111194.1 | 0.043124 |
| NDUFA6-DT | 0.016046 |
| MIR646HG | 0.028019 |
| AC145285.2 | 0.018881 |
| AC105345.1 | 0.029107 |
| LINC-PINT | 0.042027 |
| JPX | 0.021461 |
| AC012618.3 | 0.019371 |
| AL096828.1 | 0.024097 |
| AL160408.3 | 0.009606 |
| LINC01798 | 0.015875 |
| AL035701.1 | 0.045333 |
| LINC02705 | 0.036 |
| AL161618.1 | 0.016501 |
| SKINT1L | 0.044515 |
| LINC01031 | 0.000236 |
| AL683813.2 | 0.016792 |
| AP003774.2 | 0.001107 |
| AL357500.1 | 0.009012 |
| AC022613.1 | 0.01933 |
| KCP | 0.014142 |
| AC124242.1 | 0.00519 |
| AC079760.2 | 0.000612 |
| AC012213.1 | 0.015831 |
| DLGAP2 | 0.006068 |
| AP001972.3 | 0.009972 |
| LINC02704 | 0.034271 |
| AC022784.1 | 0.000425 |
| LINC02723 | 0.002963 |
| LINC01833 | 0.000215 |
| AC010931.1 | 0.040612 |
| AL035252.3 | 0.041398 |
| AC079760.1 | 0.003506 |
| LINC02265 | 0.033653 |
| AC121764.1 | 0.001471 |
| LINC02766 | 0.045982 |
| CHRNA1 | 0.028746 |
| LINC01088 | 0.046821 |
| FAM41C | 0.014727 |
| AL356421.2 | 0.016802 |
| MIR34AHG | 0.000708 |
| AC011379.2 | 0.012243 |
| LINC01863 | 0.013581 |
| LINC01312 | 8.07E-05 |
| OGFRP1 | 4.36E-06 |
| LINC00987 | 0.006057 |
| A2MP1 | 0.001652 |
| AL589745.1 | 0.008086 |
| NKAPP1 | 0.018499 |
| LINC00261 | 0.046834 |
| LINC01412 | 0.035843 |
| SEC24B-AS1 | 0.03533 |
| AP000424.1 | 0.046831 |
| AC012368.1 | 0.008326 |
| AC005082.1 | 0.011373 |
| LRRC8C-DT | 0.020728 |
| AC008957.1 | 0.01397 |
| AL035587.1 | 0.000322 |
| SOX2-OT | 0.016784 |
| HAGLR | 0.030742 |
| ZRANB2-AS2 | 0.000877 |
| SLC38A3 | 0.015914 |
| LINC01128 | 0.000391 |
| PTGDS | 0.03468 |
| NBPF1 | 0.009852 |
| LINC01484 | 0.030033 |
| HAR1A | 0.027133 |
| LINC02365 | 0.022324 |
| GMDS-DT | 0.004518 |
| AL034397.2 | 0.048548 |
| AC105942.1 | 0.039747 |
| ANKRD66 | 0.030071 |
| AC083805.1 | 0.001459 |
| CT69 | 0.006286 |
| AP001347.1 | 0.022273 |
| AP003472.1 | 0.041767 |
| CMAHP | 0.000234 |
| AL035670.1 | 0.003517 |
| LINC01913 | 0.004113 |
| LINC00460 | 0.038631 |
| AC091849.2 | 0.0054 |
| WT1-AS | 0.046419 |
| AC007255.1 | 0.003273 |
| AP004608.1 | 0.033514 |
| CDK6-AS1 | 0.040638 |
| PRKG1-AS1 | 3.56E-05 |
| AC012668.3 | 0.006716 |
| AC025871.2 | 0.005314 |
| LINC00996 | 0.004577 |
| AC090559.1 | 0.000513 |
| AC108718.1 | 0.016705 |
| LINC02657 | 0.001716 |
| PAQR9-AS1 | 0.000584 |
| FAM87A | 0.006242 |
| C5orf66 | 0.028332 |
| AC012485.1 | 0.036471 |
| LINC02198 | 0.029538 |
| LINC02572 | 0.013192 |
| CROCCP2 | 0.023799 |
| AL450311.1 | 0.035995 |
| AC093772.1 | 0.019218 |
| LINC02390 | 0.002806 |
| LINC02739 | 0.010993 |
| AC021028.1 | 0.046519 |
| SLC2A1-AS1 | 0.01075 |
| LINC01891 | 0.01399 |
| AC027117.1 | 0.016644 |
| AC064807.2 | 0.016055 |
